# CAR-T Cells for the Treatment of Central Nervous System Tumours: Known and Emerging Neurotoxicities

**DOI:** 10.3390/brainsci14121220

**Published:** 2024-11-30

**Authors:** Leonardo Palazzo, Valentina Pieri, Giulia Berzero, Massimo Filippi

**Affiliations:** 1 Neurology Unit, IRCCS Ospedale San Raffaele, 20132 Milan, Italy; palazzo.leonardo@hsr.it (L.P.); pieri.valentina@hsr.it (V.P.); filippi.massimo@hsr.it (M.F.); 2 Faculty of Medicine, Vita-Salute San Raffaele University, 20132 Milan, Italy; 3 Neurorehabilitation Unit, Neurophysiology Unit, Neuroimaging Research Unit, Institute of Experimental Neurology, Division of Neuroscience, IRCCS Ospedale San Raffaele, 20132 Milan, Italy

**Keywords:** CAR-T cells, CNS tumours, neurotoxicity, ICANS, TIAN

## Abstract

The advent of chimeric antigen receptor (CAR)-T cells has recently changed the prognosis of relapsing/refractory diffuse large B-cell lymphomas, showing response rates as high as 60 to 80%. Common toxicities reported in the pivotal clinical trials include the cytokine release syndrome (CRS) and the Immune effector Cell-Associated Neurotoxicity Syndrome (ICANS), a stereotyped encephalopathy related to myeloid cell activation and blood–brain barrier dysfunction, presenting with a distinctive cascade of dysgraphia, aphasia, disorientation, attention deficits, vigilance impairment, motor symptoms, seizures, and diffuse brain oedema. The tremendous oncological efficacy of CAR-T cells observed in systemic B-cell malignancies is leading to their growing use in patients with primary or secondary central nervous system (CNS) lymphomas and in patients with solid tumours, including several CNS cancers. Early studies conducted in adult and paediatric patients with solid CNS tumours reported a distinct profile of neurotoxicity referred to as Tumour inflammation-associated neurotoxicity (TIAN), corresponding to local inflammation at the tumour site manifesting with focal neurological deficits or mechanical complications (e.g., obstructive hydrocephalus). The present review summarises available data on the efficacy and safety of CAR-T cells for solid and haematological CNS malignancies, emphasising known and emerging phenotypes, ongoing challenges, and future perspectives.

## 1. Introduction

Over the last decade, chimeric antigen receptor (CAR)-T cells have emerged as effective cellular immunotherapies to treat relapsing/refractory haematological malignancies [1,2,3,4,5,6,7,8,9,10]. CAR-T cells consist of autologous T lymphocytes engineered ex vivo to express a synthetic fusion receptor binding a predetermined surface antigen on tumour cells. Upon intravenous infusion into the patient, CAR-T cells kill the encountered tumour cells that express the cognate antigen [11,12]. Due to their mechanism of action, CAR-T cells are associated with immune-related adverse events [13,14], including the cytokine release syndrome (CRS), a systemic inflammatory state driven by soluble mediators, and the Immune effector Cell-Associated Neurotoxicity Syndrome (ICANS), a stereotyped encephalopathy temporally and pathophysiologically related to the CRS. These adverse events, which were a safety concern in pivotal clinical trials because of their life-threatening potential, are now well classified and managed through established case definitions, grading, and treatment protocols [15,16].

To date, six commercial CAR-T cell products have been approved by regulatory agencies, including four CAR-T cell products targeting the antigen CD19 for treating relapsing/refractory B-cell malignancies (i.e., Kymriah, Yescarta, Tecartus, and Breyanzi) and two CAR-T cell products targeting the B-cell maturation antigen for treating relapsing/refractory multiple myeloma (i.e., Abecma and Carvykti). The tremendous oncological efficacy of CAR-T cells in these settings, with response rates as high as 60 to 80% [1,2,3,4,5,6,7,8,9,10,17], is now driving the development of novel constructs to expand their use [18,19,20]. Though developed for systemic lymphomas, CAR-T cells are now being increasingly used in patients with primary or secondary central nervous system (CNS) lymphoma and are on trial for a number of solid neoplasms, including lung [21,22,23], breast [24,25], prostate [26], gastrointestinal [27,28], and CNS tumours [29,30,31,32,33,34,35,36,37,38,39,40,41,42,43,44,45,46,47,48,49,50,51,52,53,54].

The administration of CAR-T cells to patients with solid CNS tumours was originally accompanied by the concern of immune-related adverse events leading to severe intrathecal inflammation and fatal complications, as anticipated in preclinical models [43]. Although, indeed, events of localised inflammation were observed in the early clinical trials [32,36,46,48,52,53], these were successfully managed with symptomatic measures, enabling clinical studies to move forward.

This review summarises current evidence on known and emerging neurotoxicities observed in patients receiving CAR-T cells for solid and haematological CNS tumours, focusing on clinical presentation and management.

## 2. The Cytokine Release Syndrome

The CRS is a very common systemic adverse event following CAR-T cell administration, affecting up to 80% of patients, with a proportion of severe cases reaching 10% in real-world series [55,56,57]. The onset of the CRS is typically within days from CAR-T cell infusion, lasting 1 to 10 days [13,58]. 

The CRS is caused by a supraphysiological proinflammatory cytokine release [14] resulting from the engagement of CAR-T cells with their target and the bystander activation of endogenous immune cells, leading to a cytokine storm, which is responsible for clinical symptoms [59,60]. The CRS usually manifests with fever (≥38 °C) and constitutional symptoms. The occurrence of hypotension and hypoxia, related to endothelial dysfunction and vascular leakage, marks the progression to severe CRS [15,16]. 

If the inflammatory response is not halted by treatment, which includes symptomatic measures (e.g., fluids, vasopressors, and respiratory support), tocilizumab (IL-6 receptor antagonist) [61,62], and corticosteroids [58,63], the CRS might result in multiorgan dysfunction and death.

## 3. The Immune Effector Cell-Associated Neurotoxicity Syndrome (ICANS)

In the pivotal clinical trials of CAR-T cells for systemic B-cell malignancies, neurotoxicity was defined and graded according to the Common Terminology Criteria for Adverse Events (CTCAE) [64] or the CARTOX [65] scoring system. The definition of ICANS by the American Society for Transplantation and Cellular Therapy (ASTCT) in 2019 [15] was met by a wide consensus, and this term has been widely used ever since in replacement of previous case definitions. 

ICANS is defined as a peculiar pattern of encephalopathy developing within days after the infusion of CAR-T cells and evolving through a stereotyped sequence of aphasia, dysgraphia, apraxia, attention deficits, vigilance impairment, motor deficits, and seizures [15,66]. If not halted, this sequence can result in diffuse cerebral oedema, coma, and patient death [15].

Real-world data indicate that ICANS occurs in 15% to 30% of patients receiving CD19 CAR-T cells [57,67,68,69]. ICANS shows a very close temporal and causal relation with the CRS, intervening 3 to 5 days following CAR-T administration [58] in patients having developed symptoms of CRS (e.g., fever, hypotension) [13]. The two conditions share a number of common predictors of occurrence and severity, including high tumour burden, higher peaks of CAR-T in the blood, and increased serum levels of proinflammatory cytokines [70,71], emphasising the existence of common pathogenetic factors.

The pathophysiology of ICANS is thought to be sustained by an exaggerated activation of the immune system promoted by the proinflammatory cytokines secreted by activated CAR-T cells and recruited myeloid cells (e.g., IL-1, IL-6, IFNγ, TNF, and nitric oxide) [13,14]. The result is an increase in the permeability of the blood–brain barrier (BBB) [60,72] that, in turn, facilitates the accumulation of CAR-T cells and proinflammatory mediators within the CNS, aggravating intrathecal inflammation and causing diffuse cerebral oedema [13,66,73].

The diagnosis of ICANS primarily relies on its stereotypical clinical presentation, although neuroimaging and/or cerebrospinal fluid (CSF) findings may be needed as supportive criteria. Besides excluding alternative causes of neurological deterioration, magnetic resonance imaging (MRI) might show, among others, focal areas of T2/FLAIR hyperintensity or diffusion restriction in the supratentorial white matter and/or the brainstem, or diffuse leptomeningeal enhancement, findings that have been associated with greater ICANS severity and poorer outcomes [66,71,72,74,75]. CSF analysis might be indicated in the event of a differential diagnosis with infectious conditions, while on ICANS, the most common—albeit nonspecific—finding is that of increased protein levels reflecting increased BBB permeability [66,72].

The management of ICANS depends on its severity [15] (Table 1), relying on high-dose corticosteroids [58,76] as a primary measure to halt the exaggerated immune response [13]. In steroid-refractory cases of ICANS, biological agents (e.g., IL-1 and/or IL-6 inhibitors) [72,77,78], dasatinib [79], and/or intrathecal chemotherapy [80,81] might be used on empirical grounds. 

Although most patients recover completely following treatment, fatal neurotoxicity is reported in about 2% of patients receiving CD19 CAR-T cells [57,72,82,83].

**Table 1:** Main clinical features and management indications for Immune effector Cell-Associated Neurotoxicity Syndrome (ICANS) and Tumour inflammation-associated neurotoxicity (TIAN) based on clinical severity.

	ICANS		TIAN	
	Main Clinical Features According to the ASTCT [15] and the EBMT/EHA [58] Consensus	Management According to the EBMT/EHA Guidelines [58]	Main Clinical Features According to an Expert Definition [84]	Management in Reported Cases [84]
**Grade 1**	Awakens spontaneously; mild disorientation in time and space; mild aphasia; mild attentive deficits or impaired handwriting; ICE score 7–9	Close monitoring; symptomatic management	Headache associated with fever, mild worsening of pre-existing neurological signs and symptoms	Observation; symptomatic management
**Grade 2**	Awakens to voice; moderate expressive and/or receptive aphasia (able to communicate with effort); impaired handwriting; apraxia; attentive deficits; ICE score 3–6	IV dexamethasone 10 mg every 6 h for 1–3 days	Moderate changes in the neurological exam that substantially affect function	Corticosteroids and/or anakinra
**Grade 3**	Awakens only to tactile stimulus; severe global aphasia (unable to communicate their needs); any focal or generalised seizure; ICE score 0–2	IV dexamethasone 10 mg every 6 h for 1–3 days; if no improvement, IV methylprednisolone 1 g per day followed by tapering; consider transfer to the ICU	Severe neurological signs and symptoms affecting critical cardiorespiratory functions; clinical signs/symptoms of increased ICP responsive to intervention	Corticosteroids and/or anakinra; CSF drainage; hyperosmolar diuretics; cardiopressor and/or positive pressure airway support (e.g., BiPAP, CPAP)
**Grade 4**	Unarousable and/or unresponsive; life-threatening, prolonged seizures; deep focal motor weakness; symptoms or signs of increased ICP; ICE score not assessable	IV methylprednisolone 1 g per day for 3 days followed by tapering; if no improvement, consider alternatives (e.g., anakinra, siltuximab, intrathecal or systemic chemotherapy); admission to the ICU indicated	Life-threatening elevated ICP refractory to initial intervention (e.g., CSF drainage); clinical signs and symptoms of impending herniation; severe brainstem dysfunction requiring endotracheal intubation	Emergent EVD or VPS placement; endotracheal intubation
**Grade 5**	Death due to ICANS	–	Death due to TIAN	–

Abbreviations: BiPAP: Bilevel positive airway pressure; CPAP: continuous positive airway pressure; CSF: cerebrospinal fluid; EBMT/EHA: European Society for Blood and Marrow Transplantation/European Haematology Association; EVD: external ventricular drain; ICANS: Immune effector Cell-Associated Neurotoxicity Syndrome; ICE score: immune effector cell encephalopathy score; ICP: intracranial pressure; ICU: intensive care unit; IV: intravenous; TIAN: Tumour inflammation-associated neurotoxicity; VPS: ventriculoperitoneal shunt.

## 4. Tumour Inflammation-Associated Neurotoxicity (TIAN)

As expected, in patients receiving CAR-T cells for CNS tumours, a pattern of neurotoxicity that did not fit into the definition of ICANS, referred to as ”Tumour inflammation-associated neurotoxicity (TIAN)”, was observed [46].

The term TIAN was first used by Majzner et al. referring to the neurological symptoms observed in their pivotal clinical trial of GD2 CAR-T cells for diffuse midline gliomas (DMGs) published in 2022 [46]. The pattern of neurotoxicity observed in the trial consisted in the transient worsening of pre-existing focal deficits and/or in the occurrence of mechanical complications related to local oedema and inflammation [46]. Consistently, brain MRI showed increased peritumoural oedema and mass effect on T2/FLAIR sequences [46,84]. The neurotoxicity was successfully managed by corticosteroids, hyperosmolar diuretics, hypertonic saline, biological agents, and CSF drainage when indicated [46]. All patients recovered following these therapeutic measures, and no neurotoxicity-related deaths occurred [46].

Subsequent clinical observations consistent with the original description by Majzner et al. led a group of experts to embrace the term TIAN to indicate this emerging neurotoxicity [84]. Two types of TIAN were distinguished: type 1, related to the mechanical effects of local inflammation and oedema, resulting in increased intracranial pressure, obstructive hydrocephalus, or brain herniation; and type 2, related to a transient immune-mediated dysfunction of local neural circuits manifesting with the worsening of pre-existing focal deficits [84]. Type 1 TIAN usually requires immediate measures, including high-dose corticosteroids, hyperosmolar therapy, or CSF diversion, while type 2 TIAN is usually manageable conservatively through observation and supportive care [84,85]. A TIAN scoring system was proposed by the expert consensus [84], although established interventional protocols and guidelines have not been redacted yet (Table 1).

**Figure 1:**
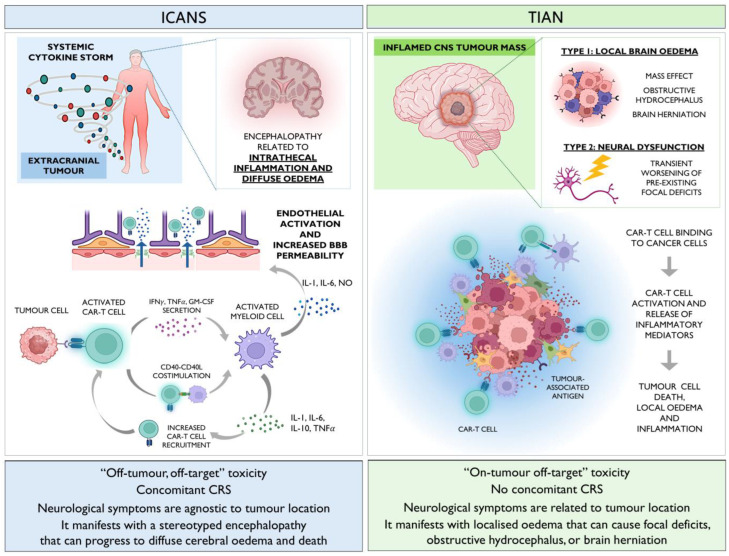
Immune effector Cell-Associated Neurotoxicity Syndrome (ICANS) and Tumour inflammation-associated neurotoxicity (TIAN): the two paradigms of CAR-T cell neurotoxicity in patients with CNS tumours. Left panel: Immune effector Cell-Associated Neurotoxicity Syndrome (ICANS) generally occurs in the context of the cytokine release syndrome (CRS). Upon binding to their target, CAR-T cells produce cytokines including IFN-*γ*, TNF*α*, and GM-CSF, promoting macrophage recruitment and activation. Macrophages are also engaged by CAR-T cells in a contact-dependent manner through CD40-CD40L costimulation. Activated macrophages secrete inflammatory mediators including IL-1, IL-6, and nitric oxide, resulting in endothelial activation and increased BBB permeability. The activated endothelium stimulates, in turn, immune cell trafficking to the CNS and intrathecal inflammation. The cytokines produced by activated myeloid cells also sustain CAR-T cell recruitment to tumour sites, creating a self-perpetuating loop. Right panel: The interaction between CAR-T cells and tumour cells at the tumour site results in localised inflammation manifesting as Tumour inflammation-associated neurotoxicity (TIAN) type 1 (local oedema possibly causing obstructive hydrocephalus or cerebral herniation) or type 2 (dysfunction of local neural circuits). Abbreviations: BBB: blood–brain barrier; CAR-T: chimeric antigen receptor T cell; CNS: central nervous system; CRS: cytokine release syndrome; GM-CSF: granulocyte macrophage colony-stimulating factor; IL: interleukin; IFN*γ*: interferon gamma; NO: nitric oxide; TNF*α*: tumour necrosis factor alpha. Created with Biorender.com.

Differently from ICANS, which is related to a systemic proinflammatory state (Figure 1, left panel), TIAN seems to result from localised inflammation at the tumour site following CAR-T cell binding to their target (Figure 1, right panel) [84]. It is therefore not surprising that, in TIAN, clinical presentation does not correspond to a stereotyped encephalopathy but mostly consists of focal deficits reflecting the neuroanatomical location of the tumour [36,46,86,87,88]. For the same reason, TIAN can be accompanied by fever, as a symptom of intrathecal inflammation, but not by other signs of CRS [84].

## 5. Neurotoxicity in Patients with CNS Lymphomas

In the early clinical trials investigating the efficacy of CD19 CAR-T cells for relapsed/refractory systemic B-cell malignancies, patients with intracranial tumour burden were excluded [1,4,5] due to concerns of unsuccessful CAR-T cell trafficking to the CNS and the fear of a potential increase in the incidence and severity of neurotoxicity. Nonetheless, as clinical experience advanced, an increasing number of patients with primary (PCNSL) or secondary (SCNSL) CNS lymphoma received CD19 CAR-T cells, following consistent observations that these products reach clinically significant intrathecal concentrations [89,90,91].

Table 2 [6,87,88,92,93,94,95,96,97,98,99,100,101,102,103,104,105,106] reports the oncological response rates and the neurotoxicity rates in the main series of PCNSL and SCNSL available to date, consisting of small-to-medium-sized retrospective/prospective cohorts of adult patients with recurrent/refractory disease. As shown in a recent meta-analysis of 30 patients with PCNSL and 98 patients with SCNSL, the overall prevalence of neurotoxicity was 53% in PCNSL (18% grade 3–4) and 48% in SCNSL (26% grade 3–4), in line with the reports of registrational studies [107]. Although neurotoxicity manifested in most cases as ICANS, with the prototypical encephalopathy accompanied by CRS and increased serum levels of proinflammatory biomarkers [6,87,88,92,93,94,95,96,97,98,99,100,101,102,103,104,105,106,107], cases of TIAN presenting with a worsening of baseline neurological deficits were also reported [87,88,97]. MRI findings reflected the known neuroradiological phenotypes of ICANS [95,106,108] and TIAN [87,88]. In some cases, the distinction between neurotoxicity and early tumour progression was challenging [87,88,94,97,106], so that some patients received additional oncological treatment in the inability to separate between the two [87,88]. Neurotoxicity was managed according to international recommendations [58], with corticosteroids as first-line agents and add-on biologics and/or intrathecal chemotherapy in refractory cases [87,88,92,93,94,95,96,97,98,99,100,101,102,103,104,105,106]. Neurotoxicity-related deaths were limited to a few isolated cases (Table 2) [87,95], suggesting a safety profile similar to patients without intracranial disease.

With the limits of the scarce data available to date, leptomeningeal involvement has been reported as a predictor of both the occurrence [105] and the severity [106] of neurotoxicity, warranting an assessment in further prospective studies. In line with previous observations in patients with B-cell lymphoma and no CNS disease [70,71], systemic tumour burden, baseline proinflammatory state, and serum levels of circulating cytokines were confirmed as relevant predictors for the subsequent occurrence of ICANS both in patients with PCNSL and patients with SCNSL [87,102,103].

The oncological efficacy of CAR-T cells was confirmed to be very high across studies, with overall response rates as high as 60 to 80% in both PSNCL and SCNSL (Table 2) [6,87,88,92,93,94,95,96,97,98,99,100,101,102,103,104,105,106].

## 6. Neurotoxicity in Patients with Solid CNS Tumours

To date, evidence on the efficacy and safety of CAR-T cells in patients with solid CNS tumours has been limited to early phase I clinical trials conducted in small populations of adult and paediatric patients with high-grade gliomas (HGGs) [29,30,32,33,34,35,36,37,38,39,40,41,46,47,48,51,52,54], anaplastic ependymomas [32,35], and anaplastic meningiomas [50].

The protocols used for solid CNS neoplasms differed from haematological malignancies with regard to a number of features, including CAR-T administration route and schedule. Intraventricular and intracavitary/intratumoural were often preferred to intravenous injections in an attempt to achieve greater intrathecal anti-tumour responses and, at the same time, reduce systemic side effects [29,30,31,32,33,34,35,36,37,38,39,40,41,46,47,48,50,51,52,53,54]. Intrathecal administration was often achieved through the use of intraventricular devices [32,34,37,46] that, despite some inherent risks, also offered the advantage of a prompt and safe CSF drainage in case of need [84]. Administration schemes often entailed multiple doses to reduce the risk of pseudoprogression and improve tolerability [32,34,35,36]. Other relevant specificities of the trials of CAR-T cells conducted in patients with solid CNS tumours concerned the population under study, which also included adolescents and children besides adults, and the adoption of the CTCAE [64] instead of the ASTCT [15] criteria to record and grade neurotoxicity.

The prevalence of neurotoxicity was high in most studies, but severe neurotoxicity was limited [29,30,31,32,33,34,35,36,37,38,39,40,41,46,47,48,50,51,52,53,54], and no neurotoxicity-related deaths were reported. The CTCAE grading system has indeed been known to overestimate the prevalence of low-grade neurological adverse events, as symptoms like mild headache, confusion, or slurred speech, scored as grade 1–2 by the CTCAE, may not meet the criteria for ICANS according to the definition of the ASTCT [16]. Interestingly, the typical ICANS phenotype was less frequently documented compared to patients with CNS lymphomas [29,30,31,32,33,34,35,36,37,38,39,40,41,46,47,48,50,51,52,53,54]. This is consistent with the notion that CRS and, to a lesser extent, ICANS are dependent on systemic inflammation, which is lower or absent in the case of solid CNS tumours [84,109]. ICANS cases were mostly observed in clinical trials that entailed the intravenous administration of CAR-T cells [39,40,46,48], while the prevalence of ICANS was low or absent in the studies that administered CAR-T cells exclusively by an intrathecal route [32,34,50,51]. The neurotoxicity pattern observed in patients with solid CNS tumours mostly included headache and worsening of pre-existing and/or appearance of new focal deficits [32,39,41,48,50,51], in line with the paradigm of TIAN [46,84]. Isolated fever was a recurrent feature of TIAN, observed in nearly all patients in some studies [46,52,53], albeit a typical CRS was uncommon. Notably, most patients developing fever had received CAR-T cells intravenously [40,46,54] rather than intrathecally. Because of this complex scenario, a revised grading system accounting for individual baseline neurological deficits and encompassing the clinical manifestations of both ICANS and TIAN, as recently used by Bagley and colleagues [53], is awaited to meet the assessment needs of patients receiving CAR-T cells for CNS neoplasms.

Several phase I clinical trials of CAR-T cells in adult and paediatric patients with HGG and/or other CNS tumours (https://www.clinicaltrials.gov/ accessed on 20 October 2024) are ongoing in the United States, Europe, and Asia at the time this review is being written (Supplementary Table S1) and should soon expand the available data on the safety profile of CAR-T cells in this clinical setting.

### 6.1. High-Grade Gliomas

Table 3 summarises the results of available studies and case reports of CAR-T cell approaches in adult and paediatric patients with HGGs (i.e., diffuse grade 3 and 4 gliomas), including H3K27M-mutant DMGs, diffuse intrinsic pontine gliomas, and glioblastomas (GBMs), all of which share a grim prognosis [110].

Most studies addressed the setting of progressive/recurrent disease [29,30,33,37,38,39,40,41,47,51,52,53,54], although few experiences of CAR-T cell administration in newly diagnosed patients, as a complement to radiotherapy [36] or in combination with radiation and temozolomide [48], were reported. Some studies were enriched with patients having multifocal disease and/or deep-seated tumours, since these characteristics limit the range of therapeutic options and confer poorer prognosis [30,39].

Several cell surface antigens have been used as CAR targets in adult and paediatric HGGs, including the IL-13 receptor alpha 2 (IL13R*α*2) [29,30,33,37,53], the human epidermal growth factor receptor 2 (HER-2) [38], the epidermal growth factor receptor variant III (EGFRvIII) [36,39,40,41,52], the GD2 ganglioside [47], the B7 homolog 3 protein (B7-H3) [50,51], and the erythropoietin-producing human hepatocellular carcinoma A2 receptor (EphA2) [54] (Table 3). These antigens were selected as overexpressed and/or selectively expressed by tumour cells [111,112,113,114], thereby limiting ‘off-tumour on-target’ toxicities. Some of these antigens had already been the target of vaccination approaches (e.g., EGFRvIII [115], IL-13R*α*2 [116]) because of this property.

Since, due to tumour heterogeneity, the use of a single target antigen raised concerns about treatment escape, recent studies have explored the efficacy and safety profile of bispecific antibody-armed [48], bivalent [53], or T-cell-engaging antibody molecule-secreting [52] CAR-T cells to enhance therapeutic results. Combining CAR-T cells with alkylating chemotherapies [48] or with immune checkpoint inhibitors [36] has been attempted as an alternative or complementary approach to improve tumour response rate and duration.

#### 6.1.1. Adult High-Grade Gliomas

Most studies in the adult population concerned GBM, the most common malignant primary brain tumour in adults and one of the deadliest cancers [110]. The therapeutic options at recurrence are thin [110], accounting for the will to investigate CAR-T cell efficacy in this setting. The neurotoxicity profile observed in adult patients with HGG typically consisted of focal neurological deficits reflecting tumour location accompanied by peritumoural inflammation on MRI [29,38,47,53], according to the paradigm of TIAN. Less frequently, the neurological presentation was compatible with a diffuse brain dysfunction resembling ICANS, manifesting with encephalopathy and/or seizures (Table 3) [36,37,52,53]. A variable percentage of cases, ranging across studies from <10% up to 70% (Table 3), met the definition for severe neurotoxicity (grade 3–4) according to the CTCAE. Fever was commonly observed [52,53], yet rarely associated with the other clinical correlates of the classic CRS. Despite initial severity, in most cases, neurological symptoms were transient, lasting from 24 h to 7 days. Treatment consisted of high-dose corticosteroids followed by slow tapering [37,41,53], with the association of biological agents (e.g., anakinra [53]) when needed. No life-threatening increase in intracranial pressure requiring surgical intervention occurred.

#### 6.1.2. Paediatric High-Grade Gliomas

CNS tumours are among the leading causes of cancer-related death in children [117]. Among those, DMGs have been the object of great interest for CAR-T cell approaches because of their surgical inaccessibility and limited response to standard cytotoxic treatments, conferring an abysmal prognosis [86]. Although initial clinical experiences were accompanied by safety concerns due to fatal cases of acute hydrocephalus observed in orthotopic mouse models of DMG [43], no neurotoxicity-related deaths were observed in patients [34,35,46]. The available data from early clinical trials [32,34,35,46] are summarised in Table 3. Fever and/or symptoms of CRS highly differed across studies that deployed different CAR-T administration routes [32,34,35,46]. Neurotoxicity reflecting the paradigm of TIAN, with focal deficits accompanied by increased peritumoural oedema and/or contrast enhancement intensification on MRI, was commonly observed, concerning nearly all cases in some small case series [32,34,46]. TIAN was generally transient and managed using corticosteroids, hypertonic saline, and biological agents [46], resolving without permanent sequelae [32,34,46]. In a few cases of severe TIAN occurring in patients with DMG, the local mass effect led to an increase in intracranial pressure requiring CSF drainage via an Ommaya reservoir, with prompt clinical benefit [46].

### 6.2. Other Adult and Paediatric Tumours

Data on solid CNS tumours other than gliomas are scarce and mostly limited to preclinical studies on medulloblastoma, ependymoma, and other rare embryonal tumours [42,45,118]. The available clinical data include two interim analyses of phase I trials conducted in children and young adults with anaplastic ependymoma treated with HER-2 [32] and IL-13R*α*2 [35] CAR-T cells administered by locoregional route. The observed neurological toxicities were consistent with TIAN and characterised by transient headache and/or worsening of pre-existing deficits, with accompanying fever and evidence of increased peritumoural oedema on brain MRI [32]. Lastly, the case of one adult patient with anaplastic meningioma recurring after multiple surgeries and radiosurgeries and receiving intracavitary B7-H3 CAR-T cells with manageable local toxicity has been reported [50].

## 7. Ongoing Challenges in Diagnosing, Managing, and Predicting Neurotoxicity

In the view of a growing use of CAR-T cells in patients with solid and haematological CNS tumours, an updated consensus on the definition and management of neurotoxicity has been advised in order to account for the specificities of this clinical setting [84]. Differently from patients with haematological malignancies and no intracranial disease, patients with CNS tumours are often burdened by baseline neurological deficits, which might complicate the assessment of acute neurotoxicity [41,53]. The local inflammation resulting from CAR-T cell activation might cause mechanical effects, including obstructive hydrocephalus and brain herniation, complications to which patients without intracranial disease were not exposed [84]. Advanced neuroimaging [119,120,121] or positron emission tomography studies [122] might be needed to distinguish between early tumour progression and neurotoxicity. All these considerations emphasise the challenges of CAR-T cell use in patients with CNS tumours and the need for a dedicated consensus on the definition, diagnosis, and management of neurotoxicity in this clinical setting.

Extensive effort is being devoted to the development of strategies to minimise the incidence and severity of CAR-T cell neurotoxicity. The pre-emptive administration of immune modulators has been advocated as a possible measure to reduce the exaggerated inflammatory response associated with the therapeutic effect of CAR-T cells. While tocilizumab did not prove effective for preventing severe neurotoxicity [123,124], prospective trials are now evaluating anakinra, an IL-1 receptor antagonist, for the prophylaxis of both CRS and ICANS (NCT04148430, NCT04359784, NCT04150913), based on evidence suggesting that IL-1 signalling is implicated in the pathogenesis of ICANS. Promising results coming from the interim results of one of those trials [125] show that prophylactic anakinra administered from day 2 to day 10 following CAR-T cell infusion results in a low incidence of severe ICANS in adult patients with systemic lymphoma.

An alternative approach under study to reduce the occurrence and the severity of neurotoxicity entails the modification of CAR-T cell constructs to downplay or halt the collateral immune cell activation triggered by the binding of CAR-T cells to their target. The development of products with reduced persistence or induced inflammation [126] or the incorporation of a suicide gene to suppress life-threatening toxicities [127] are indeed appealing approaches for CNS tumours with a deep-seated location at risk of causing obstructive hydrocephalus, such as DMGs [46]. Suicide switches exploiting an inducible caspase 9-based suicide construct (iCaspase 9) [128,129] or transgene-encoded cell-surface polypeptides [130,131] are currently under study for the targeted elimination of CAR-T cells in vivo in the case of escalating toxicity. More recently, CAR designs with reversible off/on switches controlled through the administration of small molecules [132,133], light [134], or focused ultrasound [135] are the object of early studies. Though these strategies will indeed require time before entering clinical practice, they show the most promise in improving CAR-T cell safety and further expanding their use.

Since the use of CAR-T cells for solid CNS tumours is still in its infancy, limited data exist on individual clinical and biological predictors of neurotoxicity. Besides the administration route and schedule of CAR-T cells, prior and concomitant therapies [36,48] represent another relevant point to be investigated to assess the individual risk of neurotoxicity. Brain radiotherapy and alkylating chemotherapies are often part of the first-line treatment of malignant CNS tumours [136], and their potential contribution to neurotoxicity warrants dedicated studies. The past or concomitant administration of immune checkpoint inhibitors [36] might also be a relevant factor in determining the risk of neurotoxicity, especially for patients affected with SCNSL who are more frequently exposed to these agents.

The future availability of serum and/or CSF biomarkers would indeed improve our ability to predict and monitor neurotoxicity in individual patients. Among those, liquid biopsies of cell-free DNA circulating in the CSF or plasma [137] might support the distinction between early tumour progression and pseudoprogression [138,139] with limited invasiveness, as it is in other therapeutic settings.

## 8. Conclusions and Perspectives

CAR-T cells are emerging as promising treatments for malignant CNS tumours currently lacking effective treatment options. In the early clinical trials, neurological toxicities related to the presence of the tumour within the CNS have been common but rarely life-threatening, reassuring us on the safety of this approach. Neurological toxicities corresponding to the paradigm of ICANS were primarily observed in patients with PCSNL and SCNSL receiving CAR-T cells by an intravenous route, while in patients with solid CNS tumours receiving CAR-T cells intrathecally, a local neurotoxicity with the features of TIAN prevailed. An updated consensus on the case definition, grading, and management of neurological toxicities, encompassing both ICANS and TIAN, is warranted in light of the growing use of CAR-T cells for patients with CNS neoplasms. The identification of predisposing factors and predictive biomarkers of neurotoxicity is expected to be a major advancement in improving patient selection and monitoring.

## Figures and Tables

**Table 2:** Neurotoxicity rates and oncological efficacy of commercial or in-house CD19 CAR-T cells in adult (i.e., aged 16 years or older) patients with PCNSL and/or SCNSL (only studies including ≥ 5 patients are listed).

	Reference	CAR-T Product	N	Oncological Efficacy	Criteria to Define Neurotoxicity	Median Time to Neurotoxicity (days)	Neurotoxicity, n (%)	Severe Neurotoxicity *, n (%)	Neurotoxicity-Related Deaths, n (%)
**PCNSL**	Siddiqi et al., 2021 [92]	CD19 CAR-Tn/Tmem	5	CR: 60%	CTCAE	NA	5/5 (100%)	1/5 (20%)	0 (0%)
	Alcantara et al., 2022 [93]	Tisa-cel; Axi-cel	9	ORR: 66%; CR: 56%	ASTCT	8	5/9 (56%)	2/9 (22%)	0 (0%)
	Frigault et al., 2022 [88]	Tisa-cel	12	ORR: 58%; CR: 50%	ASTCT	5	6/12 (50%)	1/12 (8%)	0 (0%)
	Karschnia et al., 2023 [87]	CD-19 CAR-T	17	^†^ ORR: 69%; CR: 40%	ASTCT	3	8/17 (44%)	3/17 (18%)	1/17 (6%)
	Choquet et al., 2024 [95]	Tisa-cel; Axi-cel	25	ORR: 76%; CR: 32%	ASTCT	5	17/25 (68%)	5/25 (20%)	1/25 (4%)
**SCNSL**	Bennani et al., 2019 [96]	Axi-cel	15	ORR: 59%	CTCAE, CARTOX	NA	13/15 (87%)	5/15 (33%)	0 (0%)
	Frigault et al., 2019 [94]	Tisa-cel	8	ORR: 50%; CR: 25%	CTCAE	NA	4/8 (50%)	0/8 (0%)	0 (0%)
	Abramson et al., 2020 [6]	Liso-cel	7	ORR: 50%; CR: 50%	CTCAE	NA	2/7 (29%)	2/7 (29%)	0 (0%)
	Ahmed et al., 2021 [98]	Axi-cel; Tisa-cel	7	CR: 86%	ASTCT	NA	3/7 (43%)	1/7 (14%)	0 (0%)
	Ghafouri et al., 2021 [99]	Axi-cel	5	ORR: 80%; CR: 60%	ASTCT	5	2/7 (40%)	2/7 (40%)	0 (0%)
	Wu et al., 2021 [100]	CD19 and CD22 CAR-T	9	ORR: 81%; CR: 54%	ASTCT	NA	3/9 (33%)	1/9 (11%)	0 (0%)
	Ayuk et al., 2022 [101]	Axi-cel; Tisa-cel	28	ORR: 64%; CR: 32%	ASTCT	5	13/28 (46%)	4/28 (14%)	0 (0%)
	Karschnia et al., 2022 [97]	CD19 CAR-T	10	ORR: 70%; CR: 60%	ASTCT	3	6/10 (60%)	3/10 (30%)	0 (0%)
	Liu et al., 2022 [102]	CD19 or CD20 CAR-T	6	ORR: 100%; CR: 57%	CARTOX	NA	0/6 (0%)	0/6 (0%)	0 (0%)
	Xue et al., 2022 [103]	CD19, CD20, or CD22 CAR-T	15	ORR: 71%; CR: 65%	ASTCT	6	6/15 (35%)	5/15 (29%)	0 (0%)
	Yuen et al., 2022 [106]	Axi-cel	14	CR: 58%	ASTCT	NA	6/14 (43%)	4/14 (29%)	0 (0%)
	Zhang et al., 2022 [104]	CD19, CD19 and CD20, or CD19/22 CAR-T	15	ORR: 73%; CR: 60%	ASTCT	<1	3/15 (20%)	1/15 (7%)	0 (0%)
	Epperla et al., 2023 [105]	CD-19 CAR-T	61	ORR: 68%; CR: 57%	ASTCT	NA	34/61 (57%)	15/61 (44%)	0 (0%)
	Karschnia et al., 2023 [87]	CD-19 CAR-T	27	^†^ ORR: 69%; CR: 40%	ASTCT	4	18/27 (67%)	4/27 (15%)	1/27 (4%)

Abbreviation: ASTCT: American Society of Transplantation and Cellular Therapy; Axi-cel: axicabtagene ciloleucel (Yescarta); CAR-T: chimeric antigen receptor T cell; CARTOX: CAR-T cell therapy-associated TOXicity; CR: complete response; CTCAE: Common Terminology Criteria for Adverse Events; Liso-cel: lisocabtagene maraleucel (Breyanzi); N: number of patients included; n: number of events; NA: not available; ORR: objective response rate; PCNSL: primary central nervous system lymphoma; SCNSL: secondary central nervous system lymphoma; Tisa-cel: Tisagenlecleucel (Kymriah); Tn/Tmem: T naïve/T memory. * Severe neurotoxicity was defined as grade 3–4 neurotoxicity. ^†^ Metrics are referred to the entire study population, comprising both patients with PCNSL and patients with SCNSL.

**Table 3:** Neurotoxicity in clinical trials of CAR-T cells in adult and paediatric high-grade (i.e., grade 3 and 4) diffuse gliomas, grouped by target antigen.

Target Antigen	Reference	Route of Admin.	N	Population	Tumour Type	Neurotoxicity ^†^, n (%)	Severe ^††^ Neurotoxicity, n (%)	Clinical Presentation of Severe Neurotoxicity	Management of Severe Neurotoxicity
**IL-13Rα2**	Brown et al., 2015 [29]	ICT	3	Adult	HGG	2/3 (67%)	2/3 (67%)	Headache; focal deficits	Steroids
	Brown et al., 2016 [30]	ICV→ICT	1	Adult	GBM	1/1 (100%)	0 (0%)	–	–
	Brown et al., 2022 [33]	ICT	6	Adult	GBM	6/6 (100%)	2/6 (33%)	Headache; encephalopathy; focal deficits	NA
	Wang et al., 2023 [35]	ICV	3	Paediatric, AYAs	H3^K27M^ DMG; H3^G34R^ HGG	4/6 (67%) ^§^	1/6 (17%) ^§^	Headache ^§^	NA
	Brown et al., 2024 [37]	ICT, ICV, dual ICV and ICT	65	Adult	HGG	NA	18/65 (28%)	Headache; encephalopathy; focal deficits; hydrocephalus	Steroids
**HER-2**	Ahmed et al., 2017 [38]	IV	17	Paediatric, adult	GBM	2/16 (13%)	0 (0%)	–	–
	Vitanza et al., 2021 [32]	ICT	1	AYAs	HGG	1/1 (100%)	0 (0%)	–	–
**EGFRvIII**	O’Rourke et al., 2017 [39]	IV	10	Adult	GBM	10/10 (100%)	4/10 (40%)	Seizures; focal deficits	Steroids; siltuximab
	Goff et al., 2019 [40]	IV	18	Adult	GBM	10/18 (56%)	1/18 (6%)	Focal deficits	Steroids
	Durgin et al., 2021 [41]	IV	1	Adult	GBM	1/1 (100%)	0 (0%)	–	–
	Bagley et al., 2023 [36]	IV	7	Adult	GBM	7/7 (100%)	4/7 (57%)	Seizures; encephalopathy; focal deficits	NA
**GD2**	Majzner et al., 2022 [46]	IV→ICV	4	Paediatric, AYAs	H3^K27M^ DMG	4/4 (100%)	3/4 (75%)	Encephalopathy; focal deficits; hydrocephalus	CSF drainage; steroids; tocilizumab; anakinra
	Liu et al., 2023 [47]	IV→ICT	8	Paediatric, adult	GBM	2/8 (25%)	1/8 (13%)	Headache	NA
**B7-H3**	Tang et al., 2021 [51]	ICT	1	Adult	GBM	1/1 (100%)	0 (0%)	_	–
	Vitanza et al., 2023 [34]	ICV	3	Paediatric, AYAs	DIPG	3/3 (100%)	1/3 (33%)	Focal deficits	None required
**EphA2**	Lin et al., 2021 [54]	IV	3	Adult	GBM	0/3 (0%)	0 (0%)	_	–
**Bispecific antibody-armed CD3 x EGFR**	Fadul et al., 2024 [48]	IV	10	Adult	HGG	10/10 (100%)	1/10 (10%)	Headache	Steroids
**Bivalent EGFR x IL-13Rα2**	Bagley et al., 2024 [53]	ICV	6	Adult	GBM	6/6 (100%)	3/6 (50%)	Encephalopathy; focal deficits	Steroids; tocilizumab; anakinra; bevacizumab
**EGFRvIII x TEAM-E**	Choi et al., 2024 [52]	ICV	3	Adult	GBM	3/3 (100%)	1/3 (33%)	Encephalopathy	Anakinra

Abbreviations: admin: administration; AYAs: adolescents and young adults; B7-H3: B 7 homolog 3 protein; CAR-T: chimeric antigen receptor T cell; CSF: cerebrospinal fluid; DIPG: diffuse intrinsic pontine glioma; DMG: diffuse midline glioma; EGFR: epidermal growth factor receptor; EGFRvIII: epidermal growth factor receptor variant III; EphA2: erythropoietin-producing hepatocellular carcinoma A2 receptor; GBM: glioblastoma; GD2: disialoganglioside D2; HER-2: human epidermal growth factor receptor 2; HGG: high-grade glioma; ICT: intracavitary/intratumoural; ICV: intracerebroventricular; IL-13Rα: interleukin 13 receptor α2; IV: intravenous; N: number of patients included; n: number of events; NA: not available; TEAM-E: T-cell-engaging antibody molecule against wild-type EGFR; IV→ICT: intravenous followed by intracavitary/intratumoural; IV→ICV: intravenous followed by intracerebroventricular; -: not applicable. ^†^ In all studies, except for Bagley et al., 2024 [53], neurotoxicity was defined and graded according to the Common Terminology Criteria for Adverse Events (CTCAE). ^††^ Severe neurotoxicity was defined as grade 3–4 neurotoxicity. ^§^ Data refer to the whole population, also including patients with ependymoma.

## Data Availability

This study did not entail the generation of new data. Data sharing does not apply to this article.

## Supplementary Materials

The following supporting information can be downloaded at: https://www.mdpi.com/article/10.3390/brainsci14121220/s1, Table S1: List of ongoing phase I clinical trials of CAR-T cells in adult and paediatric patients with primary central nervous system tumours (https://clinicaltrials.gov).

## Abbreviations

ASTCT	American Society of Transplantation and Cellular Therapy
Axi-cel	axicabtagene ciloleucel
AYAs	adolescents and young adults
BBB	blood–brain barrier
B7-H3	B 7 homolog 3 protein
CAR-T	chimeric antigen receptor T cell
CARTOX	CAR-T cell therapy-associated TOXicity
CNS	central nervous system
CR	complete response
CRS	cytokine release syndrome
CSF	cerebrospinal fluid
CTCAE	Common Terminology Criteria for Adverse Events
DIPG	diffuse intrinsic pontine glioma
DMG	diffuse midline glioma
EGFR	epidermal growth factor receptor
EGFRvIII	epidermal growth factor receptor variant III
EphA2	erythropoietin-producing hepatocellular carcinoma A2 receptor
GBM	glioblastoma
GD2	disialoganglioside D2
GM-CSF	granulocyte macrophage colony-stimulating factor
HER-2	human epidermal growth factor receptor 2
HGG	high-grade glioma
ICANS	Immune effector Cell-Associated Neurotoxicity Syndrome
ICT	intracavitary/intratumoural
ICV	intracerebroventricular
IFN*γ*	interferon gamma
IL	interleukin
IV	intravenous
Liso-cel	lisocabtagene maraleucel
MRI	magnetic resonance imaging
NA	not available
NO	nitric oxide
ORR	objective response rate
PCNSL	primary central nervous system lymphoma
SCNSL	secondary central nervous system lymphoma
TEAM-E	T-cell-engaging antibody molecule against wild-type epidermal growth factor receptor
TIAN	Tumour inflammation-associated neurotoxicity
Tisa-cel	Tisagenlecleucel
TNFα	tumour necrosis factor alpha
Tn/Tmem	T naïve/T memory
