# Supplementary material for: CAR-T Cells for the Treatment of Central Nervous System Tumours: Known and Emerging Neurotoxicities
Source: Brain Sci. 2024 Nov 30;14(12):1220. doi: 10.3390/brainsci14121220 (PMC11727498; doi:10.3390/brainsci14121220)
Supplement: Supplementary file 1 [file brainsci-14-01220-s001.zip › brainsci-3319923-supplementary.pdf]

Supplementary Table S1. List of ongoing phase I clinical trials of CAR-T cells in adult and paediatric patients with primary central nervous system tumours (https://clinicaltrials.gov).

Clinicaltrial.gov identifier	Trial location	CAR-T cell target antigen	Route of admin.	Population	Tumour type	Clinical setting
NCT04185038	USA	B7-H3	ICT and/or ICV	Paediatric; AYAs	CNS tumours	Newly diagnosed or recurrent
NCT05835687	USA	B7-H3	ICT and/or ICV	Paediatric; AYAs	CNS tumours	Newly diagnosed or recurrent
NCT06221553	Thailand	B7-H3	ICV	Paediatric	DIPG	Newly diagnosed or recurrent
NCT05474378	USA	B7-H3	ICT and/or ICV	Adult	GBM	Recurrent
NCT05366179	USA	B7-H3	ICV	Adult	GBM	Recurrent
NCT04385173	China	B7-H3	ICT and/or ICV	Adult	GBM	Recurrent
NCT04077866	China	B7-H3	ICT and/or ICV	Adult	GBM	Recurrent
NCT05241392	China	B7-H3	ICT and/or ICV	Adult	GBM	Recurrent
NCT06482905	China	B7-H3	ICT and/or ICV	Adult	HGG	Recurrent
NCT04661384	USA	IL-13Rα2	ICV	Adult	CNS tumours	Recurrent
NCT04003649	USA	IL-13Rα2	ICT and/or ICV	Adult	GBM	Recurrent
NCT04510051	USA	IL-13Rα2	ICV	Paediatric; AYAs	CNS tumours	Recurrent
NCT02208362	USA	IL-13Rα2	ICT and/or ICV	Adult; Paediatric	HGG	Recurrent
NCT05298995	Italy	GD2	IV	Paediatric; AYAs	CNS tumours	Recurrent
NCT04196413	USA	GD2	IV and ICV	Adult; Paediatric	DIPG and other DMG	Newly diagnosed or recurrent
NCT04099797	USA	GD2	IV and ICV	Paediatric; AYAs	CNS tumours	Newly diagnosed or recurrent
NCT03500991	USA	HER-2	ICT and/or ICV	Paediatric; AYAs	CNS tumours	Recurrent
NCT02442297	USA	HER-2	ICT	Adult; Paediatric	CNS tumours	Recurrent
NCT03638167	USA	EGFR806	ICT and/or ICV	Paediatric; AYAs	CNS tumours	Recurrent
NCT05802693	China	EGFRvIII	ICT	Adult	GBM	Recurrent
NCT05063682	Finland; India	EGFRvIII	ICV	Adult	GBM	Recurrent
NCT04214392	USA	CLTX	ICT and/or ICV	Adult	GBM	Recurrent
NCT05353530	USA	CD70	IV	Adult	GBM	Recurrent
NCT05577091	China	CD44 x CD133	ICT and/or ICV	Adult	GBM	Recurrent
NCT06186401	USA	IL-13Rα2 x EphA2	IV	Adult	GBM	Newly diagnosed or recurrent
NCT05660369	USA	EGFRvIII x TEAM-E	ICV	Adult	GBM	Newly diagnosed or recurrent
NCT05168423	USA	IL-13Rα2 x EGFR	ICV	Adult	GBM	Recurrent
NCT05768880	USA	B7-H3 x EGFR x HER-2 x IL-13Rα2	ICV	Paediatric; AYAs	CNS tumours	Newly diagnosed or recurrent
NCT05868083	China	EGFR x EGFRvIII x HER2 x IL-13Rα2	IV	Adult	GBM	Recurrent

Abbreviations: admin.: administration; AYAs: adolescent and young adults; B7-H3; B7 homolog 3 protein; CD: cluster of differentiation; CLTX: chlorotoxin tumour-targeting domain; CNS: central nervous system; DIPG: diffuse intrinsic pontine glioma; EGFR: epidermal growth factor receptor; EGFRvIII: epidermal growth factor receptor variant III; EphA2: erythropoietin-producing hepatocellular carcinoma A2 receptor; GBM: glioblastoma; GD2: glycosphingolipid disialoganglioside 2; HER-2: human epidermal growth factor receptor 2; HGG: high-grade glioma; IL-13Rα2: interleukin-13 receptor alpha 2; ICT: intracavitary/intratumoural; ICV: intracerebroventricular; IV: intravenous; TEAM-E: T-cell-engaging antibody molecule against wild-type EGFR; USA: United States of America.
